# Identifying factors relevant in the assessment of return-to-work efforts in employees on long-term sickness absence due to chronic low back pain: a focus group study

**DOI:** 10.1186/1471-2458-12-77

**Published:** 2012-01-24

**Authors:** Anna Muijzer, Jan H Geertzen, Wout E de Boer, Johan W Groothoff, Sandra Brouwer

**Affiliations:** 1 Department of Health Sciences, Community and Occupational Medicine, University Medical Center Groningen, University of Groningen, Groningen, The Netherlands; 2 Department of Rehabilitation Medicine, Center for Rehabilitation, University Medical Center Groningen, University of Groningen, Groningen, The Netherlands; 3 Academy of Swiss Insurance Medicine, University Medical Centre, University of Basel, Basel, Switzerland

## Abstract

**Background:**

Efforts undertaken during the return to work (RTW) process need to be sufficient to prevent unnecessary applications for disability benefits. The purpose of this study was to identify factors relevant to RTW Effort Sufficiency (RTW-ES) in cases of sick-listed employees with chronic low back pain (CLBP).

**Methods:**

Using focus groups consisting of Labor Experts (LE's) working at the Dutch Social Insurance Institute, arguments and underlying grounds relevant to the assessment of RTW-ES were investigated. Factors were collected and categorized using the International Classification of Functioning, Disability and Health (ICF model).

**Results:**

Two focus groups yielded 19 factors, of which 12 are categorized in the ICF model under activities (e.g. functional capacity) and in the personal (e.g. age, tenure) and environmental domain (e.g. employer-employee relationship). The remaining 7 factors are categorized under intervention, job accommodation and measures.

**Conclusions:**

This focus group study shows that 19 factors may be relevant to RTW-ES in sick-listed employees with CLBP. Providing these results to professionals assessing RTW-ES might contribute to a more transparent and systematic approach. Considering the importance of the quality of the RTW process, optimizing the RTW-ES assessment is essential.

## Background

Chronic low back pain (CLBP) is an important cause of work disability and sickness absence [1,2]. In European countries, up to 35% of work disability is caused by CLBP [2]. In the Netherlands, the total costs of disability because of back pain were estimated at 1361 million Euros in 2007, which comprises a proportion of 38.5% of the total costs of back pain [3]. An effective return to work (RTW) process is essential to prevent applications for disability benefits due to chronic disability [4,5]. With the high number of disabled workers, the outcome and content of this RTW process are important issues [5-8]. Although the assessment of the RTW process is part of the application of disability benefits in several countries (i.e. Denmark, Germany, the Netherlands, Norway) [8], few studies focus on this assessment or on the factors relevant to the quality assessment of the RTW process [4,5].

The RTW process can be assessed by means of the assessment of RTW Effort Sufficiency (RTW-ES), as part of the evaluation of the RTW process in relation to the application for disability benefits [8]. RTW efforts made in the RTW process include all activities undertaken by employee, employer or health professionals involved in the RTW process to improve the work ability of the sick-listed employee in the period between onset of sickness absence and the application for disability benefits [9]. The perspective of this assessment is that if the RTW process is designed effectively and the RTW efforts are sufficient, the chances of RTW have been tested in an optimal way, and RTW should be achieved in accordance with health status and work ability of the sick-listed employee [10]. The assessment of RTW-ES investigates the quality of the RTW process. Assessing RTW-ES is of importance when considering the remaining functional possibilities of the employee and determining future RTW opportunities.

In the Netherlands, this assessment takes place prior to the assessment of functional and earning capacity (i.e. the income that would be generated if the individual would be employed to full functional capacity) as part of the disability evaluation, after two years of sickness absence [9]. The RTW-ES assessment is performed only when the Dutch employee has not fully returned to work after two years of sickness absence, but does have remaining work ability and is applying for disability benefits. If the RTW efforts are not considered sufficient, the application for disability benefits can be delayed to make sure that the necessary efforts can still be undertaken. This is similar to the consequences in other countries, where the rehabilitation period is extended (i.e. Denmark) or a rehabilitation subsidy is applied for (i.e. Finland, Germany) [8]. The assessment is based on a reintegration report, which is written by both employer and employee. The reintegration report includes a problem analysis, i.e. a mandatory description of the (dis)abilities of the employee made by an Occupational Physician (OP) of the Occupational Health Service (OHS) hired by the employer, an action plan, i.e. the plan designed to achieve work resumption, and the employee's opinion regarding the RTW process. Records of all interventions, intermittent RTW process advice by independent professionals, and agreements between employer and employee are also required in the reintegration report [8,9,11].

The assessment of RTW-ES in the Netherlands is performed by Labor Experts (LE's) of the Dutch Social Insurance Institute (SII). LE's are specialized in the field of vocational rehabilitation and after graduating, have followed a one to two year intensive post academic in company training. LE's assess whether all opportunities for RTW have been examined and undertaken by the employee or employer, if applicable. The LE's also focus on the context of the RTW process, i.e. factors which might influence the RTW process and its quality, like the relationship between employer and employee, and the employee's attitude. The LE's consider only the non-medical aspects of the RTW process, but they can consult a Social Insurance Physician (SIP) about the medical aspects of the RTW process, e.g. medical interventions and medical prospects. If necessary, the LE's can consult the employee, employer or OP to gather or verify information.

If the efforts made during the RTW process are considered insufficient by the LE, the direct and indirect consequences can be serious [8]. A direct consequence of insufficient efforts is that the application for disability benefits is delayed for a maximum of one year, or until the employer and/or employee have undertaken the necessary actions. A more indirect consequence is that insufficient efforts are an indication that the time to RTW of the employee has been unnecessarily prolonged. In 2010, LE's of the Dutch SII's were responsible for over 27,000 RTW-ES assessments [12].

Over the last years protocols and guidelines have been developed for professionals to improve the quality and standardize their decision-making process [13-15]. These protocols are systematically developed and contain recommendations based on evidence from published literature. In current practice, for LE's, only a protocol is available which focus mainly on procedural matters [8], and its contents have not been gathered by means of scientific evidence. Moreover, it does not provide a set of factors relevant in the RTW-ES assessment based on scientific evidence. Gathering information about the relevant factors in the assessment of RTW-ES by means of research and including this kind of evidence-based information in the existing protocol will optimize not only the transparency and reliability but also the validity of the assessment [13].

The quality and effect of the RTW process on RTW outcome is influenced by a large number of factors [1,7,16], which makes the operationalization of 'sufficiency of RTW efforts' and the quality assessment of the RTW process a unique challenge. The assessment of RTW-ES is a complex decision making process, in which relevant factors are regarded implicitly [8]. Knowing which factors are related to RTW-ES is essential, but no guidelines as to which factors are relevant to the decision are available in the Netherlands or in other countries. Literature concerning factors relevant to the assessment of RTW-ES is scarce [8]. Also, it is of interest to know whether factors relevant to the assessment of RTW-ES can be fitted within the model of Functioning, Disability and Health (ICF) model [17]. By analyzing our results within the ICF model we aim to use a comprehensive framework. Using this well-known categorization system also facilitates the connection to existing and future literature. This way, our approach could help to improve comparability.

A possible source of information about factors relevant to the assessment of RTW-ES is the implicit knowledge of the professionals performing the assessment. Focus group research is a suitable method to gather information on a decision process which is otherwise performed implicitly by professionals [18,19]. The focus group process aims to explore and clarify individual and shared perspectives [18,20,21]. This is particularly effective in complex processes [21], such as the assessment of RTW-ES. The method to unravel the assessment is to gather arguments for the assessment outcome in a standardized setting, and to identify the underlying grounds, thereby making the knowledge and experience of the professionals more explicit [18,19,22]. These underlying grounds are necessary to understand the translation of gathered information into arguments used for the decision, a different conclusion of professionals may arise in identical cases because different grounds are being referred to [19,23].

The main aim of this study was to identify the factors relevant to RTW-ES by means of focus groups, by investigating arguments and underlying grounds relevant to the assessment of RTW-ES in cases of sick-listed employees with CLBP, and to categorize these factors within the ICF model.

## Methods

### Focus groups

**Figure 1:**
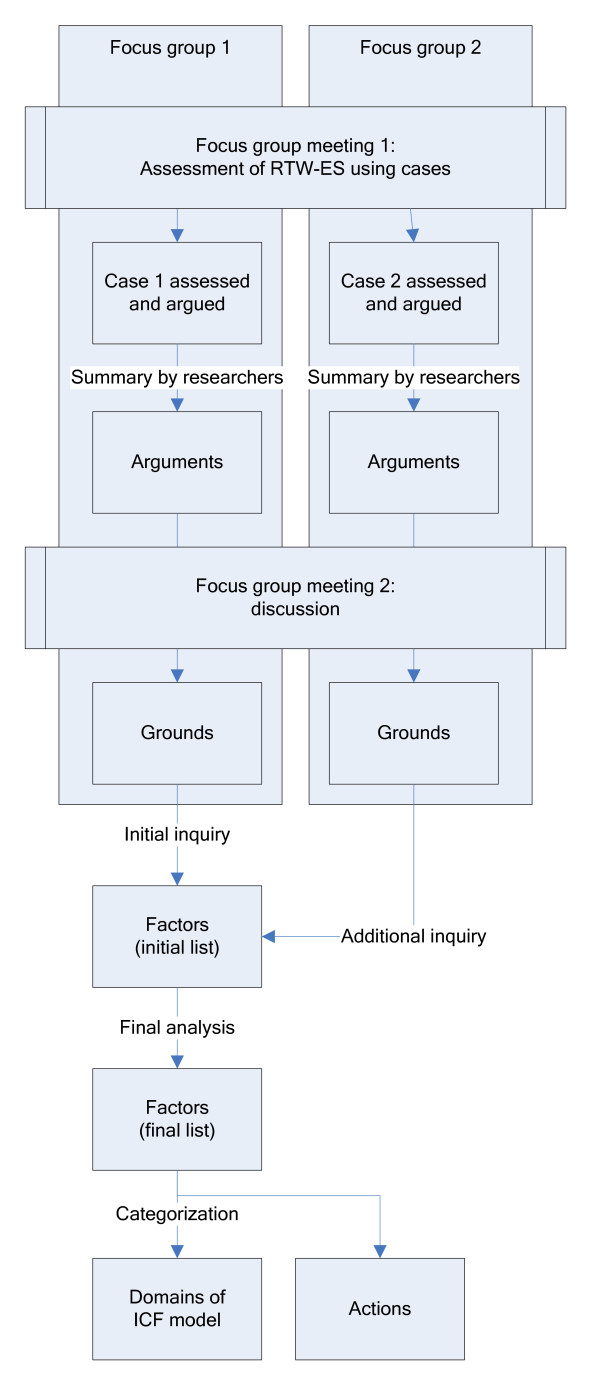
**Overview of procedure to make factors relevant to Return-to-work Effort Sufficiency explicit by means of focus groups**.

The focus groups consisted of LE's working at the SII in the Netherlands, We aimed at bringing together two focus groups of 5-8 LE's. A minimum of five LE's in each group is necessary to ensure response diversity, and a maximum of eight LE's to facilitate discussion later in the focus group process. A total of 32 LE's were contacted by SII staff members, 16 from SII's in the northern region, 16 from SII in the central region of the Netherlands. LE's were selected by the staff members for their expertise in the assessment of RTW-ES, and to include members of all SII offices of the region. If they agreed, the researcher (AM) contacted them and explained the study, and asked for their participation. Each focus group had two meetings, where two cases of RTW-ES were introduced. Both focus groups assessed a different case. The results from the second focus group were used to confirm and add to the findings of the first focus group. The procedure will be described in detail below (see also Figure 1).

### Cases

The cases were selected by two of the authors (AM and SB), with the help of five LE's. These LE's acted as an expert group and did not participate in the focus groups. The two cases were selected to reflect two realistic situations on the basis of a well-defined RTW process and outcome, resemblance to daily practice for the assessors, and sufficiency of the information in the reintegration report. The cases represented employees on sickness absence for two years due to CLBP. The employees had not returned to work fully, showed no comorbidity (e.g. other diseases causing or prolonging the sickness absence), and had been available for RTW interventions (e.g. had not been institutionalized for a prolonged period of time).

The first case was about a 50 year old female with secondary vocational education, working in healthcare. The employee had been working in a large company as a facility management worker. The work ability assessment by the occupational physician revealed that the employee was no longer suitable for her original job, and that she had a restriction in working hours (a maximum of 26 h a week, and no evenings or nights). There was no chance of improvement in work ability. The company could not offer any suitable work, except for a temporary job. There had been a medical exam, but no RTW expert or agency had been put into action. Furthermore, there was a conflict between employer and employee, originating from a disagreement about each other's efforts.

The second case was about a 56 year old male with secondary vocational education, working in public transportation. The work ability assessment by the occupational physician revealed that the employee could not return to his own work fully, but could return to other (more suitable) work fully. The employer could not offer a full-time suitable job, but could offer a combination of suitable tasks, allowing the employee to RTW fully. The employee, however, insisted on returning to his own work, resulting in a partial RTW only. The employer had consulted experts from the SII, which advised a professional approach concerning CLBP interventions. These interventions did not take place.

### Assessment

#### First focus group meeting - collecting the arguments

During the first meeting, a group of LE's was asked to assess individually the RTW efforts in the case presented. The procedure used matched the standard procedure at the Dutch SII, in which the LE receives the report made in the RTW process and the instruction to assess the RTW efforts. During the assessment, the LE's had access to their usual sources of information (e.g. legislation, guidelines, etc.). They were not allowed to consult each other or other LE colleagues. The LE's were given the opportunity to contact a fictitious employee, employer, physician of the OHS, and a physician of the SII. These roles were all performed by LE's, who had prepared their roles and had contacted physicians for advice and further information if they played the role of physician. This standard procedure closely resembles the standard procedure used in the Dutch SII's when assessing RTW-ES.

In addition, the LE's received a clear instruction on the procedures of the day, and were asked to answer two questions. The questions were aimed at gathering information about 1) arguments used for deciding about the sufficiency of the RTW efforts, and 2) the decision outcome (sufficiency of RTW efforts). In order to analyze the data gathered per case, the authors (AM and SB) made an inventory of the arguments mentioned by the LE's, and also gathered information about who mentioned each argument. In order to collect the underlying grounds behind the arguments, a second meeting took place.

#### Second meeting - collecting the underlying grounds

During the second meeting, the LE's were invited to participate in a discussion session. This meeting took about four hours, and was chaired by a (senior) LE, with assistance of two of the authors (AM and SB). During the meeting, the participants were asked to explain why their arguments are relevant to the assessment of RTW efforts, thereby revealing the underlying ground of the argument. The ground is the underlying reason for mentioning the argument, and will make knowledge and experience more explicit. The other participants were asked if they agreed with each ground provided, and were then asked to provide other grounds if available. The grounds were discussed and altered if necessary until all focus group members agreed.

The researchers analyzed the grounds produced in the first focus group and collected factors from these grounds. All the words mentioned in the grounds have been considered, thereby collecting aspects relevant to the assessment of RTW-ES. Next, the grounds from the second focus group were analyzed to confirm factors found in the first focus group and identify additional factors. Finally, the authors (AM and SB) discussed all factors with each other in order to identify universal phrasing and correspondence between the factors. If different terms were used to describe the same factor, the terms would be combined (i.e. 'limitations in terms of hours', 'severity of limitations' and 'energy' were all filed under 'functional capacity'). If AM and SB did not agree on the phrasing, other authors (JHBG and JWG) were consulted.

The factors were then categorized in accordance with the International Classification of Functioning, Disability and Health (ICF) model (WHO), into five domains of the assessment: 1) functions 2) activities, 3) participation, 4) personal, and 5) environmental [17].

### Ethics and consent

According to the Dutch Medical Research Involving Human Subjects Act (WMO), approval is not necessary for this focus group study. The professionals' opinions were collected with their consent, without any requirement to follow altered rules of behavior. No real patients were involved in the study, and the anonymized, altered cases used in this study were made available by the SII [24].

## Results

The first focus group consisted of eight LE's, of which seven attended both meetings. The second focus group consisted of seven LE's, of which five attended both meetings. The reasons for absence were a pre-existing appointment in one case, and illness in two cases. Seven out of twelve LE's were male, and the LE's had between two and six years of experience in assessing RTW-ES.

### Arguments and grounds

During the first meeting, the members of the first focus groups each assessed the case assigned to their focus group. The authors (AM and SB) summarized 42 arguments. An example of such an argument is 'a reorganization has taken place', which was mentioned by three LE's. During the second meeting, these three LE's were asked to elaborate on the underlying grounds, which was in this case 'a reorganization limits the availability of work'. The other focus group members were asked if they agreed with the ground, and if not, to alter the ground to achieve agreement. The final ground in this case was 'a reorganization might limit the availability of suitable work'. In this manner 48 underlying grounds were collected. The members of the second focus group provided 28 arguments in total, from which 38 underlying grounds were collected. Consensus was reached on all grounds.

**Figure 2:**
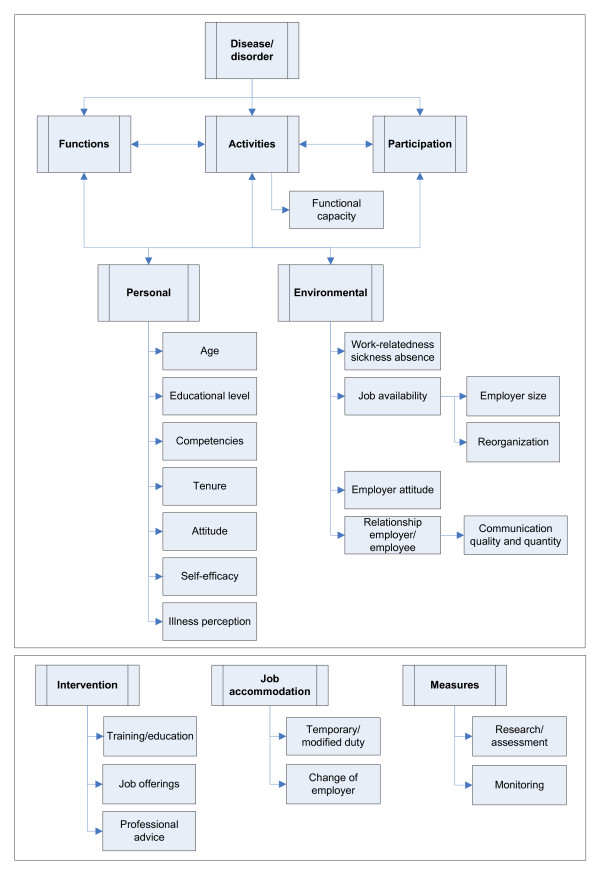
**Factors relevant to Return-to-work Effort Sufficiency according to focus groups, in relation to the International Classification of Functioning, Disability and Health (ICF) model**.

In order to collect factors relevant to RTW-ES, the authors (AM and SB) analyzed the grounds gathered in the first focus group. This initial inquiry produced 46 factors, in which considerable overlap was present. For example, 'limitations in terms of hours', 'severity of limitations' and 'energy' were all filed under 'functional capacity'. Investigation of the grounds provided by the second focus group provided an additional 12 factors. These 58 factors were taken into consideration when performing the final analysis. This final analysis yielded 19 factors, of which 12 could be filed under regular domains of the ICF model (activities, personal and environmental domain) (see Figure 2). Seven factors are technically actions, and could not be fitted in the ICF model. An example of this is 'training', which is related to the assessment of RTW-ES, but has an impact on the domain of personal factors (e.g. educational level, competencies), environmental factors (e.g. job availability). In our categorization, 'training' is not considered in terms of available services, but in terms of an actual intervention performed and supported by the stakeholders, which might have an effect on several factors related to RTW-ES. Nineteen factors are described below in relation to the ICF model, including examples of grounds mentioned in relation to these factors (see also table 1).

**Table 1:** Examples of grounds for factors relevant to RTW-ES according to focus groups

Factor	Ground example
**ICF domains**	

**Activities**	

Functional capacity	"The type and severity of limitations determine the suitability for work"

**Personal**	

Age	"As the age of the employee increases, the chances of RTW with a different employer decreases"

Educational level	"The educational level is an indicator of the possibilities to RTW"

Competencies	"The competencies of an employee are important because of job availability"

Tenure	"The longer the tenure, the more can be expected of the current employer"

Attitude	"A positive attitude of the employee has a positive influence on RTW"

Self-efficacy	"A low self-efficacy increases the chance of non-RTW"

Illness perception	"A difference between limitations which are experienced and work ability hinders RTW"

**Environmental**	

Work-relatedness of sickness absence	"If the sickness absence is caused by work, the employer's obligations towards the employee increase"

Job availability	"The number of available jobs determines the chance of RTW"

*Employer size*	"The larger the company size, the more opportunities for the employee to RTW"

*Reorganization*	"A reorganization is at the cost of the number of available jobs and the chance of RTW"

Employer's attitude	"A positive attitude of the employer towards RTW opportunities increases RTW"

Relationship employer/employee	"The relationship between employer and employee determines the readiness of the employee to cooperate with the RTW process"

*Communication quality and quantity*	"A good communication between employer and employee increases the chance of RTW"

**Actions**	

**Intervention**	

Training/education	"The employer should consider requests for training by the employee"

Job offerings	"The chance of RTW is increased by job offerings of the employer"

Professional advice	"Advice of a professional can be helpful if the progress in the RTW process is slower than expected"

**Job accommodation**	

Temporary/modified duty	"Sustained, durable work is preferred over temporary work"

Change of employer	"If RTW with the current employer is not likely, RTW with a different employer should be investigated"

**Measures**	

Assessment	"The employer should investigate the availability of suitable work within the company"

Monitoring	"The progress of the RTW process should be monitored by the employer"

### Activities

#### Functional capacity

Related to the category 'activities' of the ICF model is 'functional capacity'. The focus group participants stated that functional capacity is relevant to the assessment of RTW-ES, in that it determines which activities the employee can undertake and which opportunities for participation are remaining. Functional capacity refers to the capability of performing tasks and activities [14]. Examples of grounds mentioned in relation to functional capacity are "The type and severity of limitations determine the suitability for work", "The limitations indicate whether the original job is no longer suitable" and, "The limitations determine the chance of RTW".

### Personal domain

In relation to the personal domain of the ICF model, seven factors have been found, which are described in detail below.

#### Age

Age is a relevant factor of the assessment of RTW-ES. The LE's stated that"As the age of the employee increases, the chances of RTW with a different employer decreases".

#### Educational level

Also important to the assessment of RTW-ES is educational level, because "The educational level is an indicator of the possibilities to RTW".

#### Competencies

The competencies of the employee are relevant to the assessment of RTW-ES ("The competencies of an employee are relevant to RTW").

#### Tenure

The tenure of the employee, or the number of years employed by the current employer, is mentioned as a relevant factor to RTW-ES ("The longer the tenure, the more can be expected of the current employer").

#### Attitude

Also mentioned is the attitude of the employee, the employee's like or dislike towards an aspect (e.g. RTW goal, activity, capacity). This attitude plays an important role in the effectivity of the RTW process and an optimal outcome. A ground mentioned is "A positive attitude of the employee has a positive influence on RTW", and "The attitude of the employee towards RTW should be positive to promote RTW".

#### Self-efficacy

Self-efficacy is about the employee's beliefs about his or her capability to produce effects (e.g. perform a certain behavior or reach a certain goal) [25]. This factor is mentioned by the LE's as relevant to RTW-ES: "A low self-efficacy increases the chance of non-RTW", and "Not working while having work ability decreases the self-efficacy of the employee".

#### Illness perception

is relevant to RTW-ES as the perception of the employee towards the disability and the consequences: "A difference between limitations which are experienced and work ability hinders RTW".

### Environmental domain

Four factors are filed under the environmental domain of the ICF model, and are described below.

#### Work-relatedness of sickness absence

A factor relevant to the assessment of RTW-ES mentioned by the focus group attendees was whether the sickness absence was work-related. LE's stated: "If the sickness absence is caused by work, the employer's obligations towards the employee increase".

#### Job availability

Also relevant to RTW-ES is job availability, whether suitable jobs are available to the employee with regard to the employee's work ability. A ground mentioned in relation to job availability is for example "The number of available jobs determines the chance of RTW". Specifically mentioned as relevant to job availability are employer size ("The larger the company size, the more opportunities there are for the employee to RTW"), and reorganization ("A reorganization is at the expense of the number of available jobs and the chance of RTW").

#### Employer's attitude

The attitude of the employer as relevant to the assessment of RTW-ES ("A positive attitude of the employer towards RTW opportunities increases RTW").

#### Relationship employer/employee

Also relevant to the assessment of RTW-ES is the relationship between employer and employee (or supervisor and employee). This influences the attitude of both employer and employee, and plays an important role in the RTW process and because of that in the assessment of RTW-ES. LE's stated that "The relationship between employer and employee determines the readiness of the employee to cooperate with the RTW process", and "The work-related relationships determine the chances of RTW". A factor related to the relationship between employer and employee is communication. The quality and quantity of communication between employer and employee is relevant to RTW-ES. The ground related to this factor is "A good communication between employer and employee increases the chance of RTW".

### Actions

The seven factors which could not be filed in the ICF model are factors which describe actions rather than a situation. These factors are categorized under interventions, job accommodation and measures.

#### Interventions

Actions relevant to the assessment of RTW-ES and categorized under interventions are training/education, job offerings and professional advice.

##### Training/education

Whether training or education is facilitated by the employer is relevant to RTW-ES. Grounds mentioned were: "The employer should consider requests for training by the employee", and "If the employer can offer work after a short training, both the training and the work should be offered".

##### Job offerings

Relevant to RTW-ES is whether the employer offers available jobs to the sick-listed employee. Grounds are for example: "The chance of RTW is increased by job offerings of the employer", and "If a suitable job is available, this job should be offered".

##### Professional advice

Requesting and following professional advice is an important factor in RTW-ES. Relevant grounds are: "Ignoring professional advice can have a negative influence on RTW outcome", and "Professional advice can be requested if there are any doubts on the prognosis of the employee".

#### Job accommodation

Factors relevant to the assessment of RTW-ES and related to job accommodation are temporary/modified duty, and the focus on a change of employer.

##### Temporary/modified duty

Facilitating and accepting temporary or modified duty is relevant to the assessment of RTW-ES. Grounds mentioned in relation to temporary or modified duty are "Sustained, durable work is preferred over temporary work", and "The employer should offer modified work to the sick-listed employee".

##### Change of employer

Also relevant to the assessment of RTW-ES is whether a change of employer is investigated and facilitated: "If RTW with the current employer is not likely, RTW with a different employer should be investigated", and "If the chances of RTW with a different employer are small, the RTW process should emphasize on RTW with the original employer".

#### Measures

Measures are defined as ways to gather information related to the RTW process. Actions related to measures which are relevant to RTW-ES are monitoring, and assessment.

##### Monitoring

Monitoring or guidance is an important effort and relevant to the assessment of RTW-ES. "The monitoring of the progress of the employee should be sufficient to achieve optimal RTW", and "Monitoring can prevent stagnation of the RTW process".

##### Assessment

An important factor is doing assessments. These assessments can for example be focused on assessing the abilities of the employee, the suitability of the available jobs the workplace: "Assessment of the work ability of the employee is essential to determining the RTW process", and "The employer should investigate the availability of suitable work".

## Discussion

Nineteen factors related to RTW-ES were identified after analyzing arguments and grounds of LE's derived from two CLBP cases. Twelve of these 19 factors can be fitted within a single domain of the ICF model. The factor functional capacity is related to 'activities'. Factors in the personal domain related to RTW-ES include age, educational level, competencies, tenure, attitude, self-efficacy and illness perception. Factors in the environmental domain related to RTW-ES are work-relatedness of the sickness absence, job availability, the relation between employer and employee, and employer's attitude. The remaining seven factors can not be fitted within the ICF model. These factors are categorized under intervention (i.e. training/education, job offerings, professional advice), job accommodation (i.e. temporary/modified duty, change of employer), and measures (i.e. assessment, monitoring).

To compare our results with other studies with regard to generalization, no literature about the relation between factors found in research on RTW and factors related to the assessment of RTW-ES was available [4]. We decided to compare the factors found in this study to the existing literature on factors related to RTW to investigate consistencies and differences between the factors related to these outcomes.

The 19 factors found to be relevant to RTW-ES in CLBP patients in this study are mostly consistent with literature on RTW. For example, the relation between a higher age, educational level and attitude of the employee and RTW has also been found in literature [26,27], however, the interpretation and direction of the relevant factor can be different when considering RTW-ES. Literature concerning RTW in patients with CLBP states that the remaining functional capacity is strongly related to RTW after sickness absence [27]. It can be assumed that this is also a reason to take functional capacity into account when assessing RTW-ES. Fewer efforts can be undertaken when an employee with limited capacity is involved. If the employee has limited remaining capacity, efforts to RTW could be considered less useful. Nevertheless, it can also be assumed that more efforts should be undertaken to promote RTW of employees with limited functional capacity, as it will be harder for them to RTW. Another example is the effort of offering temporary or modified work. Research on RTW in CLBP has found that RTW increases the well-being of the sick-listed employee [16], and that temporary work shortens the time to RTW [28,29]. Literature has also shown that the lack of modified work is related to the transition from acute to chronic LBP [30], and the availability of modified work might therefore be relevant when the effort sufficiency during the RTW process is assessed after two years.

According to LE's, investigating and offering temporary or modified work is related to RTW-ES, but they state that non-temporary work is preferred over temporary work.

When considering RTW-related outcomes, both RTW and RTW-ES can be of interest to the RTW process [4,15], but the literature of RTW can not simply be transcribed to RTW-ES. For example, undertaking an effort (e.g. offering training or education) can be considered essential to RTW-ES because it influences factors relevant to RTW-ES (positive attitude of the employer, self-efficacy of the employee), regardless of whether the training has proven to be effective to RTW. In our previous research we have examined the strength and relevance of factors related to RTW-ES and RTW among employees applying for disability benefits after 2 years of sickness absence [4], and have investigated the comparability of the factors related to these two outcomes. We have concluded that different factors are relevant to RTW-ES and RTW, but the relationship between employer and employee is relevant to both. The lack of similarity between these outcomes can be explained by the relative independence of the outcomes. For example, RTW-ES can be sufficient or insufficient, regardless of RTW outcome. For example, when the RTW outcome is sufficient, the RTW efforts are assumed to be sufficient as well. However, RTW outcome can be sufficient despite lack of RTW-ES, and in cases where RTW efforts are sufficient the RTW outcome can be negative.

A strength of this study is that this is the first study that explores the implicit knowledge used by professionals to assess RTW-ES. Using a focus group method has proven to be an intensive but effective method to collect the implicit knowledge of LE's. In order to gather a wide range of arguments, grounds and factors, two focus groups have been assembled, each using a different case. Moreover, to ensure the quality of the results, we have used a method to collect arguments which was as close as possible to being a natural situation while maintaining standardization. This way, the arguments collected by each LE could be used for group-wise discussion.

Another strength lies in the universal phrasing of the grounds mentioned by the LE's and the factors derived from these grounds. Discussions focused mostly on the applicability of the ground, i.e. if the ground could apply to all imaginable cases. For example, reorganization might not in all cases limit job availability for the sick-listed employee. Of course, some grounds (e.g. regarding the responsibilities of the employer) can be viewed in context: Dutch legislation requires the employer to undertake all efforts necessary to promote the RTW of the employee. However, these efforts are not specified, and mostly the procedural aspects and the relation between RTW efforts and RTW results are described in detail. Moreover, efforts to promote RTW are beneficial to RTW regardless of the legislatory consequences (financial or otherwise). A good employer-employee relationship is beneficial to RTW [7] and is important to RTW-ES regardless of whether the employer will experience financial consequences.

Also of interest when considering efforts relevant to RTW-ES are the assessability (possibilities for discussion) and modifiability (possibilities of alteration) of factors. For example, no assessment is necessary for the factor age, which is also not modifiable. Self-efficacy, however, is a factor which is open to discussion and should be assessed by a professional, and is also modifiable.

A limitation might be that only two cases concerning CLBP were used. Using more cases or different cases might have yielded more factors. However, we feel that by selecting two cases which each concerned CLBP, but with different backgrounds and RTW processes, we have enhanced the opportunity to gather different arguments and discuss factors in an effective way. Furthermore, LE's from two Dutch SII's were included in the study. We do not know whether these LE's are representative of their occupational group. Future studies are necessary to reproduce and expand our findings. Another limitation related to the focus group method might be our use of actors for the roles of several stakeholders. However, our priority was to provide a standardized but realistic situation, which we feel we have achieved by training these actors to portray each stakeholder.

A further point of discussion might be that no factors related to 'disease', 'functions', and participation were mentioned by the LE's. The lack of factors fitted within these categories can be attributed to the Dutch context, where disease and functions are investigated by the Social Insurance Physician (SIP) and other medical specialists. LE's mainly consider the participation as an outcome, and investigate aspects related to activities, taking personal and environmental factors into consideration when assessing RTW-ES. Also, some factors that are relevant to RTW have not been mentioned by LE's (e.g. gender, work requirements, family support), and have not been discussed.

Furthermore, by categorizing the factors derived from the focus group study in the ICF model an attempt was made to provide a clear overview and improve comparability. The categorization of the factors related to actions (e.g. measures, intervention or accommodation) was subject of debate. The ICF model is used to classify components of functioning and disability, while the actions are focused on changing one or more of these components. Moreover, using the ICF model for actions is a complicated process, and requires reduction of actions into a series of observations which could be categorized in the ICF model. [14]. The availability of training would be a factor related to the environmental domain, but the offering of the training and the effect of the training has an effect on several components (e.g. educational level, competencies, attitude and job availability).

The relevance of this study lies in that it is one of the first studies to investigate factors relevant to RTW-ES [4]. The results found in this focus group study will provide an overview of factors relevant to the assessment of RTW-ES. The assessment of RTW-ES will remain a unique and multifactorial decision making process performed by a professional (i.e. the LE) based on the information which is available and the context it is placed in (e.g. legislation). However, providing factors relevant to the assessment of RTW-ES to the professionals who perform this assessment might make the assessment more evidence-based and could contribute to a more systematic approach of the assessment of RTW-ES.

Further research is required to investigate whether the results of this study can be replicated within a different context (e.g. another country, different focus group members), and whether they are relevant in cases where the patient has a disease other than CLBP (e.g. depression), or in cases where the patient has diagnosed comorbidity. In this study, the relevance of factors has been investigated, but no distinction has been made on the association itself, e.g. whether older or younger age is relevant to RTW-ES, and in what way. Further research could elaborate on the direction of the association. Also of interest to further research is whether the professionals all consider these factors during a similar assessment, and whether the interpretation (e.g. the importance of a factor in a specific case) is comparable. It should be investigated whether the introduction of the results found in this study (i.e. an evidence-based protocol) will contribute to a more systematic approach by the professionals assessing RTW-ES. Also, if professionals have access to similar information for the assessment of RTW-ES, this could benefit the reliability of the assessment and the argumentation used in the decision-making process.

## Conclusions

In conclusion, this focus group study shows that 19 factors may be relevant to RTW-ES in sick-listed employees with CLBP. These factors fit into three domains of the ICF model (activities, personal and environmental), and also include actions which do not fit within the ICF model. Further research is necessary to replicate these findings in different contexts (e.g. case, assessor, country). Providing the results of this focus group study to professionals assessing RTW-ES might contribute to a more reliable and systematic approach. Considering the importance of the quality of the RTW process, optimizing the assessment of RTW-ES is essential.

## Competing interests

This study was funded by a SIG grant (Stichting Instituut Gak), the Netherlands. The authors declare no competing interests.

## Authors' contributions

AM carried out the study and has drafted the manuscript. JHBG participated in the design of the study and helped to draft the manuscript. WELDB participated in the design of the study and helped to draft the manuscript. JWG participated in the design of the study and helped to draft the manuscript. SB helped carry out the study, participated in its design and coordination, and helped to draft the manuscript. All authors read and approved the final manuscript.

## Pre-publication history

The pre-publication history for this paper can be accessed here:

http://www.biomedcentral.com/1471-2458/12/77/prepub
